# Evaluation of the Effects of an Immune-Boosting Food Supplement on the Severity and Frequency of Pediatric Respiratory Tract Infections: A Randomized, Double-Blind, Placebo-Controlled Clinical Trial

**DOI:** 10.3390/children13030428

**Published:** 2026-03-20

**Authors:** Fabrizio Calapai, Ilaria Ammendolia, Carmen Mannucci, Giorgia Bulferi, Lara Pauletto, Heide De Togni, Rita La Paglia, Floriana Raso, Mariaconcetta Currò, Gioacchino Calapai

**Affiliations:** 1 Department of Clinical and Experimental Medicine, University of Messina, 98166 Messina, Italy; 2 Department of Biomedical, Dental, Morphological and Functional Imaging Sciences, University of Messina, 98125 Messina, Italy; cmannucci@unime.it; 3 Department of Chemical, Biological, Pharmaceutical and Environmental Sciences, University of Messina, 98166 Messina, Italy; 4 Regulatory and Scientific Affairs Department, Schwabe Pharma Italia, 39044 Egna, Italy; giorgia.bulferi@schwabe.it (G.B.); heide.detogni@schwabe.it (H.D.T.); 5 Italian National Health Service, Azienda Sanitaria Provinciale 5, 98123 Messina, Italy; 6 Associazione Farmaceutici Industria, 20149 Milano, Italy

**Keywords:** respiratory tract infections, children, vitamins, immune defense, *Sambucus nigra*, *Malpighia glabra*, *Lactobacillus rhamnosus*

## Abstract

**Highlights:**

**What are the main findings?**

- The food supplement significantly reduced the number of respiratory tract infection (RTI) episodes in children aged 3–10 years compared with the placebo.
- The supplement also reduced RTI severity, as shown by fewer illness days, fewer days with fever, cough, and rhinitis, and reduced use of antipyretics and antibiotics, with good tolerability.

**What are the implications of the main findings?**

- Supplementation with a combination of vitamins, minerals, herbal extracts, and *Lactobacillus rhamnosus* CRL1505 may be an effective preventive strategy for reducing both the frequency and severity of pediatric RTIs.
- This approach could help lower healthcare use and the socioeconomic burden associated with recurrent RTIs in children.

**Abstract:**

**Background**: Respiratory tract infections (RTIs) are common in children and represent one of the main reasons for pediatric consultations. Although generally benign, pediatric RTIs can lead to medical complications and significant socioeconomic burden. The objective of this trial was to evaluate the efficacy of a food supplement intended to support the immune system in reducing the rate and severity of pediatric RTIs. **Methods**: A randomized, double-blind, placebo-controlled, parallel-group clinical trial was conducted to assess the efficacy and safety of a food supplement based on vitamins, minerals, herbal extracts, and *Lactobacillus rhamnosus* CRL1505 (Pegaso^®^ Immuno Junior). A daily dose was administered for approximately 60 days within a three-month period to children aged 3–10 years with at least four RTI episodes in the previous year. RTI frequency, illness days, days with fever, cough, and rhinitis, and antipyretic and antibiotic use per episode were recorded over four months. **Results**: A total of 110 children completed the study. Compared with the placebo, the active supplement significantly reduced the mean number of RTI episodes per child (2.41 ± 0.84 vs. 4.13 ± 1.66; RR = 0.745, 95% CI 0.583–0.953) as well as the number of illness days, days with fever, cough, and rhinitis, and days with antipyretic or antibiotic use. The supplement was well-tolerated. **Conclusions**: The patented supplementation based on vitamins, minerals, herbal extracts, and *Lactobacillus rhamnosus* CRL1505 (Pegaso^®^ Immuno Junior), taken over a three-month period, may reduce the frequency and severity of RTIs in children aged 3–10 years.

## 1. Introduction

Respiratory tract infections (RTIs) occur frequently in children and infants, and they are one of the main reasons for physician consultations of pediatric patients [1]. Pediatric RTIs are generally benign and resolve without treatment in a few days; however, they can produce a socioeconomic burden for children and their families and lead to medical complications [2]. Furthermore, intermittent respiratory tract infections during the early years of life have an impact on the broncho-alveolar and the maturing lung. The immune system evolves and reaches maturity during the pediatric age, and thus infections contracted in this period of life, characterized by greater vulnerability, may negatively influence bronchial and lung physiology [3].

Risk factors favoring the development of frequent RTIs are exposure to pollutants, including smoking, short duration or absence of breastfeeding together with genetic issues, and immunological deficiency [4,5]. In the first months and in the subsequent years of life, where the “immunological memory” has not yet fully developed [6], RTIs occur frequently. According to the World Health Organization (WHO), in the first five years of life, children experience four to eight respiratory tract infections per year, affecting mainly the lower respiratory tract. Respiratory infections are considered “recurrent” if there are three episodes of acute infections during a six-month period [7].

Nutrition plays a key role in strengthening the immune system [8]. The interaction between nutrition and the immune system is very complex. The immune response is compromised if nutrition is qualitatively or quantitatively insufficient, thus predisposing people to infections [9]. In particular, at every stage of the immune response, specific micronutrients, including vitamins and minerals, play a fundamental and often synergistic role, and deficiency of just one essential nutrient can compromise immunity [10]. In these cases, where a healthy diet and correct lifestyle need support, the use of natural substances with an immunostimulant action can be useful [11]. The product of this trial is a food supplement designed to support healthy immune defense in children. It contains an innovative complex based on zinc, vitamins B6, B12, and D, inulin, *Sambucus nigra*, *Malpighia glabra*, and *Lactobacillus rhamnosus* CRL1505. The immunomodulatory effect of this product was demonstrated in an in vitro model in human monocytes (THP-1) and ex vivo human peripheral blood mononuclear cells [12]. The authors concluded that combining vitamins, selected probiotics, and herbal products with immunomodulatory properties is an interesting strategy to act at different levels of the immune response.

This clinical trial was designed to evaluate the efficacy and safety of a food supplement in decreasing susceptibility to respiratory infections in children aged 3 to 10 with more than four respiratory tract infections occurring in the previous year.

## 2. Materials and Methods

Participants in this trial were enrolled in the pediatric outpatient department of the Azienda Ospedaliera Provinciale of Messina in collaboration with the Azienda Ospedaliera Universitaria (AOU) Policlinico “G. Martino” of Messina, Italy. They were recruited according to the following inclusion criteria: children of both sexes aged 3–10 years with a medical history characterized by more than four respiratory tract infections in the previous year. Exclusion criteria included the occurrence of acute upper respiratory tract infection or other infection within 7 days prior to enrolment, cystic fibrosis, immunodeficiency syndromes, abnormalities in respiratory tract anatomy, and use of immunostimulant drugs or immunosuppressants in the previous 4 weeks. At the enrolment visit before randomization, parents were informed about the trial by a pediatrician before signing the informed consent form for children to participate in this trial. Data on parents’ education and their smoking habits were collected.

After screening, children suitable for the trial according to the inclusion and exclusion criteria were randomly allocated to one of the two treatment groups. Random allocation was performed in a blinded manner where pediatricians were not aware of the treatment assignment, as described below. By a member of the CRO not involved in patient care, treatment allocation, or data capture, a randomization list was generated through randomization software using 1:1 allocation with blocks of 2 and 4. Block size was withheld from investigators. The randomization list allocated both treatments to the anticipated number of patients and a 5-digit random code for each patient. By members of the CRO not involved in patient care, treatment allocation, or data capture, verum or placebo packs were bagged in identical opaque and sealed containers labelled with the 5-digit random codes only. At enrolment and after checking inclusion and exclusion criteria, pediatricians handed out one of the remaining sealed containers to the patient’s parents. The appearance, consistency, organoleptic characteristics, and viscosity of verum and the placebo (Table 1) were indistinguishable. Both participants (their parents) and investigators were blinded regarding the interventions, and group membership was disclosed only after the analysis of the results.

Each of the two treatment regimens, verum and placebo, was administered for three months as follows: a first phase (first month) of continuous intake for 30 days of verum or placebo (one vial a day); phase 2 (second consecutive month), characterized by 15 days of no intake followed by 15 days of continuous intake of verum or placebo (one vial a day); and phase 3 (third consecutive month), characterized by first 15 days of no intake followed during the remaining 15 days by continuous intake of verum or placebo (one vial a day). Three months of the treatment period were followed by a fourth phase with 30 days of no intake for both groups of children.

The effectiveness of the treatment was evaluated through two scheduled visits at enrolment and 4 months later. The parents/caregivers used a diary to report all events affecting the respiratory tract. Parents were also asked to record in the diary the presence of symptoms such as fever, runny nose, and cough and use of antipyretic or antibiotic agents. Parents or caregivers were asked to communicate to pediatricians the occurrence of symptoms of upper respiratory tract infection to confirm the existence of the acute episode of respiratory illness. The illness episode was recorded only after pediatrician’s confirmation.

The safety of the product was evaluated by recording adverse events. Occurrence of adverse events (AEs) was observed throughout the whole clinical trial. AEs were defined as inappropriate medical events that were or were not associated with the procedures or the product.

The trial was approved by the Interagency Ethics Committee of Messina located at AOU Policlinico “G. Martino” through protocol number 32/22 on 13 September 2022. The trial was conducted according to the ethical principles of the Declaration of Helsinki and registered at ClinicalTrial.gov under the identifier NCT06218225.

### Sample Size and Statistical Analysis

A sample size of at least 110 children was calculated to demonstrate an average difference of at least one respiratory tract episode during 4 months of observation between the active treatment group and the placebo group, with a power of 80% and a significance level of 5%.

The primary outcomes were the evaluation of the number and the severity of infectious events affecting the respiratory tract [13]. A respiratory tract infection (RTI) episode was defined as an acute, self-limiting (usually within 7–10 days) illness caused by pathogens infecting the upper or lower respiratory tract. Symptoms include coughing, sore throat, congestion, or fever. The means ± standard deviation (SD) and 95% confidence intervals (CI) were calculated. The Mann–Whitney test was used to evaluate verum–placebo differences for each parameter. Demographic data are reported as means and SD. The number of RTI episodes was calculated per child, and the mean number of illness days, days with fever, cough, and rhinitis, and days with antipyretics and antibiotics were calculated per episode for each child. These data were also analyzed separately in preschool (3–6 years, 38 verum and 37 placebo) and school age (7–10 years, 18 verum and 17 placebo) children to verify the existence of differences in the effects of the product between these two subgroups.

The occurrence of episodes of RTIs is reported as the relative risk (RR), along with 95% confidence intervals (CI) and *p*-values.

A *p*-value less than 0.05 was considered statistically significant. All statistics were calculated with SPSS 29.0 software (SPSS Inc., Chicago, IL, USA).

Estimated effect sizes were determined using Cohen’s d, which provides a measure of effect size weighted according to the relative size of each sample. Cohen’s d is generally used to estimate the effect size when comparing means, and it does not change with sample size. According to Cohen, a small size effect is equal or lesser than 0.2; a medium size effect is about 0.5; and larger size effects are greater than 0.8 [14].

## 3. Results

**Figure 1:**
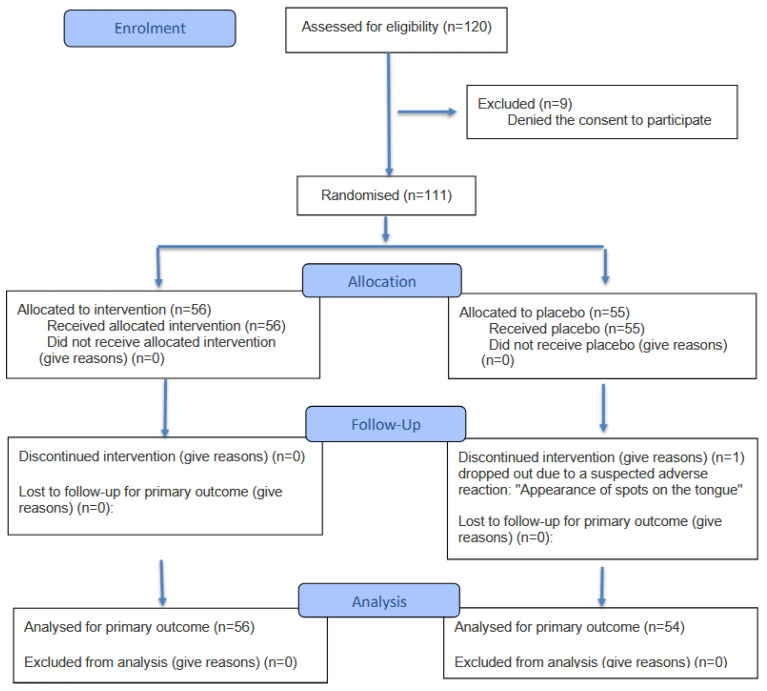
Study flowchart.

**Figure 2:**
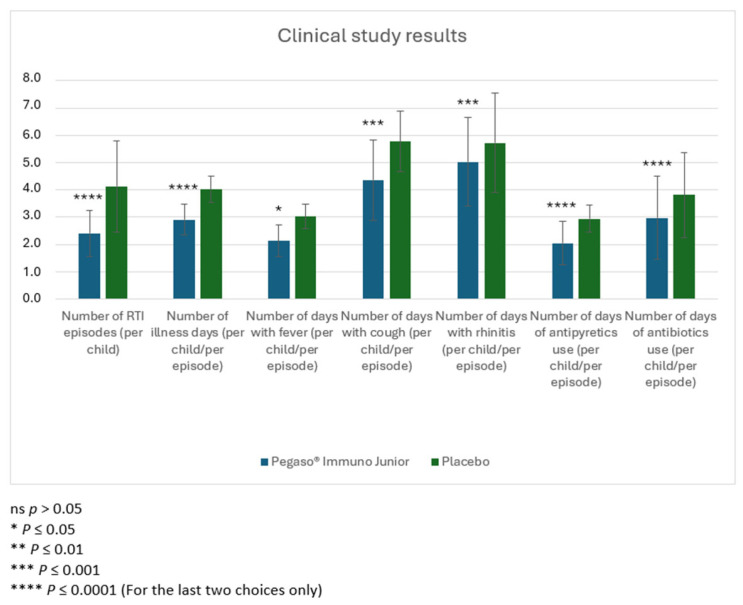
RTI episodes per child and days with symptoms or medication use per child per episode over four months. Results are presented as mean numbers and standard deviation. * *p* ≤ 0.05, ** *p* ≤ 0.01, *** *p* ≤ 0.001, **** *p* ≤ 0.0001; Mann–Whitney test for comparison of verum vs. placebo.

For the trial, 120 children were considered eligible. Parents of 111 children provided informed consent, and these children were enrolled into the study; parents of 9 children denied consent to participate. For the 110 children who completed the study (1 child dropped out), 56 children were previously randomly allocated to the group receiving the verum and 55 were previously allocated to the group receiving the placebo. Among these, 30 and 28 children in the verum and placebo groups were males, respectively, and 26 and 27 were females in the verum and placebo groups (Figure 1). Baseline characteristics of the children were comparable between both groups (Table 2). Statistically significant lower numbers of RTI episodes as well as number of illness days, days with fever, cough, and rhinitis, and days with antipyretics and antibiotics per episode were observed in the group treated with verum (Table 3, Figure 2). These differences corresponded to large effect sizes for the reduction of the number of RTI episodes, number of illness days, number of days with fever and cough, and days of antipyretic use. A significant medium-size effect was detected for the number of days with rhinitis and the number of days of antibiotic use (Table 3). In children aged 3–6 years old, the number of RTI episodes, number of illness days, days with fever, cough, and rhinitis, and days with antipyretics and antibiotics per episode were significantly reduced in the group treated with verum, with a satisfactory size effect for all of these parameters (Table 4). In children aged 7–10 years old, numbers of RTI episodes, number of illness days, days with cough, and days with antipyretics and antibiotics per episode were significantly reduced in the group treated with verum, showing a large effect size (Table 5).

Table 6 shows the relative risks for one RTI episode, more than two RTI episodes, and more than three RTI episodes during the four-month period of the trial.

One 6-year-old female patient from the placebo group dropped out due to a suspected adverse reaction: “Appearance of spots on the tongue”. The tongue stains disappeared after a few days. Rechallenge was not performed. This phenomenon was not observed in other children enrolled in the study. Therefore, it was not possible to establish a link with the product being tested (Table 7).

## 4. Discussion

Acute respiratory tract infections are among the leading cause of morbidity and mortality worldwide. The WHO evaluates that seasonal influenza causes 3–5 million cases of serious diseases requiring hospitalization and 290,000–650,000 deaths every year [15].

Analysis of results obtained from this clinical trial shows that oral intake of a food supplement (verum) reduces the incidence of RTIs and their severity in children. Reduction of incidence is shown by the lower number of RTI episodes in children treated with the product in comparison to the group of children treated with the placebo. Minor severity is revealed by the reduction of different parameters related to the single episodes (number of illness days, number of days with fever, cough, and rhinitis, number of days in which antipyretics and antibiotics were taken) as they occurred in children treated with the trial product. The reduction in antibiotic use contributes significantly to the reduction of antibiotic resistance, a phenomenon of growing importance in public health. Inappropriate use of antibiotics can lead to a risk of adverse events, alteration of the intestinal and respiratory microbiota, and a negative impact on the sustainability of healthcare systems.

Verum is a food supplement designed to modulate immune defenses in children and contains a complex based on zinc, vitamins B6, B12, and D, inulin, elderberry, *Malpighia glabra*, and *Lactobacillus rhamnosus* CRL1505. It has a significant positive effect on the incidence and severity of RTIs in children aged 3 to 10 years. Beneficial effects were shown both in preschool age and school age, with a more marked efficacy in preschool age. The sample of children recruited for this research was composed of subjects considered susceptible to RTIs because they had several RTI events in the previous year greater than four episodes.

Beneficial effects of the product investigated in the present trial are related to the biological activities of its ingredients. *Malpighia glabra* is a plant native to South America and Central America that has antioxidant and antifungal properties, commonly known as Acerola. This plant contains a high content of vitamin C, and the fruit also contains amino acids, phenolic compounds, including anthocyanins and flavonoids, and carotenoids [16]. Extracts and bioactive compounds derived from *Malpighia glabra* were shown to have antioxidant, anti-inflammatory, antitumor, antihyperglycemic, and skin protecting effects [17,18].

*Sambucus nigra* L. (Elder) and derived products have been shown to counteract flu symptoms through their immunomodulatory and antiviral effects [19]. *Sambucus nigra* L. has a long ethnobotanical history of use in the treatment of viral infections in many cultures; it is considered safe, and it is widely used in the world [20]. Proof of elderberry extracts’ safety is reflected by Food and Drug Administration approval in the United States [21]. Elderberry and acerola promote the body’s natural defenses [22].

Vitamin B6 is necessary for the health and proper functioning of the human body. It comprises a group of water-soluble chemical compounds, such as pyridoxal, pyridoxamine, pyridoxine, and their 50-phosphates. The active form, which is pyridoxal phosphate, serves as a cofactor for approximately 160 biological reactions that occur in the human body [23]. Vitamin B6 deficiency can contribute to the development of immune disorders and consequent rise in infections. The European Food Safety Authority estimates vitamin B6, together with other micronutrients, to be essential for the regular function of the immune system [24].

Vitamin B12 (cobalamin) is a water-soluble vitamin. Its active forms have a role in the methylation and synthesis of DNA, proteins, and lipids [25]. In the immune system, vitamin B12 influences lymphocyte count and the activity of natural cells killer (NK). Moreover, low levels of this vitamin lead to a decrease in the absolute number of CD4+ and CD8+ lymphocytes, increase the CD4/CD8 ratio, and reduce the activity of NK cells [26]. Furthermore, changes in a certain number of lymphocytes and in the activity of NK cells have been found with deficiency of vitamin B12 [27,28]. Vitamin B12 has a primary role in the body’s defense against viral infections. Its supplementation has been proposed as an adjuvant treatment in patients affected by COVID-19, especially in the case of patients with deficiency or deficiency risk for this vitamin [29].

Vitamin D is also involved in the regulation of the immune system [30]. Vitamin D deficiency has been linked to an increased risk of infections of the respiratory tract, including SARS-CoV-2 [31,32]. The innate immune system is the first line of defense against invading pathogens, such as viruses, which initiate the response to inflammation and activate the adaptive arm of the immune defense mechanism [32]. Because chronic activation of innate immune response can be deleterious, the maintenance of normal levels of vitamin D can be important because this substance limits the excessive production of tumor necrosis factor-α and interleukin-12, modulating the activity of the innate immune system [33]. A meta-analysis of data from 25 randomized controlled trials on vitamin D supplementation revealed a protective effect against acute respiratory infections [34].

Zinc has an essential role in the immune response. Supplementation with zinc is considered a safe and economical way to support the optimal function of the immune system, having the potential to reduce the risk and consequences of infections, including viral respiratory infections [35]. Zinc and vitamins B6, B12, C, and D contribute to the normal function of the immune system [36]. *Lactobacillus rhamnosus* CRL1505 is a probiotic favoring the balance of microorganisms inside of the intestinal microbiota [37]. Several studies have focused on probiotic microorganisms to stimulate the immune system (immunobiotics) to improve the defenses of the respiratory tract [38]. It has been shown that orally administered probiotics, including *Lactobacillus rhamnosus* CRL1505, can stimulate immunity against respiratory infections [39,40]. *Lactobacillus rhamnosus* CRL1505 modulation of the immune system seems to be mediated by Toll-like receptors in the respiratory tract and increases resistance to respiratory syncytial virus infection [41]. The strain CRL1505 has been used successfully to reduce the incidence and morbidity of viral airway infections in children [42,43].

It has been also shown that supplementation with the oligosaccharide inulin may have a protective role in respiratory tract infections in infants and children [44], and delta-inulin microparticles may increase antibody titers produced by vaccination [45].

A recent study demonstrated the combinatory mode of action of Pegaso^®^ Immuno Junior on immunomodulation in an in vitro model [12]. In conclusion, the authors observed the beneficial results of synergistic activity between probiotics and specific botanicals acting at different levels of the immune response.

## 5. Conclusions

The product used in this trial (Pegaso^®^ Immuno Junior) is a food supplement designed to support healthy immune defense in children based on scientific literature reporting beneficial effects of its ingredients in stimulating immune defense. Despite some limitations of the present trial, such as being a single-center trial and the small size of the sample, results show that oral intake of this product for approximately 60 days spread over a 3-month period was associated with a lower number of respiratory tract infections; the clinical study, however, lasted a total of 4 months. As a consequence, it is possible to hypothesize the utility of this product in lowering susceptibility to acute respiratory infections in children.

Moreover, larger and multicenter studies with biomarker-supported endpoints would help to confirm these results.

## Figures and Tables

**Table 1:** Verum and placebo composition (quantity in 1 vial).

Ingredient	Verum	Placebo
Solid phase		
Inulin	160 mg	0
*L. rhamnosus* CRL 1505	1 billion CFU	0
Vitamin B6	0.9 mg	0
Vitamin B12	1.6 µg	0
Vitamin D3	15 µg	0
Magnesium stearate	2 mg	2 mg
Maltodextrins	0	198 mg
Liquid phase		
Water	q.b. 10 mL	q.b. 10 mL
Fructose	1 mL	1 mL
*Sambucus nigra* L.	300 mg	0
*Malpighia glabra* L.	120 mg	0
Zinc	2 mg	0
Potassium sorbate	0.01 mL	0.01 mL
Sodium benzoate	0.01 mL	0.01 mL
Flavor	0.01 mL	0.01 mL
Citric acid	q.b. pH 4.00–5.00	q.b. pH 4.00–5.00

**Table 2:** Baseline characteristics of children analyzed (n = 111).

Sex	Total	Verum	Placebo
Male	58	30	28
Female	53	26	27
Age in years	5.68 ± 2.06	5.56 ± 2.17	5.69 ± 1.90
Range of age (years)	3–10	3–10	3–10
Median age (years)	5	5	5
Parent education (years)	11.97 ± 3.07	11.96 ± 3.08	11.98 ± 3.06
Passive smoking (at least one parent smoking or using electronic cigarettes)	26/111	13/56	13/55

Age and parent education are expressed as means ± standard deviation.

**Table 3:** Number of respiratory tract infections (RTIs) per child, number of illness days, number of days with fever, number of days with cough, and number of days of antipyretic use and antibiotic use per child/per episode during the 4-month period of the trial.

	Verum (n = 56)	Placebo (n = 54)	Effect Size (Cohen’s *d*)	*p*-Value
Number of RTI episodes (per child)	2.41 ± 0.84	4.13 ± 1.66	1.3	0.00001
Number of illness days (per child/per episode)	2.91 ± 0.57	4.02 ± 0.49	1.2	0.00001
Number of days with fever (per child/per episode)	2.14 ± 0.57	3.02 ± 0.45	1.7	0.00236
Number of days with cough (per child/per episode)	4.36 ± 1.48	5.77 ± 1.11	1.1	0.00001
Number of days with rhinitis (per child/per episode)	5.02± 1.62	5.72± 1.82	0.4	0.0005
Number of days of antipyretic use (per child/per episode)	2.05 ± 0.80	2.95 ± 0.50	1.3	0.00001
Number of days of antibiotic use (per child/per episode)	2.98 ± 1.53	3.81 ± 1.56	0.5	0.00008

Data are expressed as means ± standard deviation. *p*-values for Mann–Whitney test for comparison of verum vs. placebo.

**Table 4:** Number of respiratory tract infections (RTIs) per 3–6-year-old child, number of illness days, number of days with fever, number of days with cough, and number of days of antipyretic use and antibiotic use per child/per episode during the 4-month period of the trial.

	Verum (n = 38)	Placebo (n = 37)	Effect Size (Cohen’s *d*)	*p*-Value
Number of RTI episodes (per child)	2.31 ± 0.90	4.13 ± 1.62	1.4	0.000002
Number of illness days (per child/per episode)	2.89 ± 0.61	4.03 ± 0.50	2.0	0.000002
Number of days with fever (per child/per episode)	2.79 ± 1.50	3.25 ± 0.86	0.4	0.003896
Number of days with cough (per child/per episode)	4.32 ± 1.44	5.66 ± 1.10	1.1	0.000007
Number of days with rhinitis (per child/per episode)	4.84 ± 1.65	5.67± 1.90	0.5	0.000751
Number of days of antipyretic use (per child/per episode)	2.29 ± 1.34	3.33 ± 1.05	0.9	0.000009
Number of days of antibiotic use (per child/per episode)	2.87 ± 1.63	3.92 ± 1.48	0.7	0.000125

Data are expressed as means ± standard deviation. *p*-values for Mann–Whitney test for comparison of verum vs. placebo.

**Table 5:** Number of respiratory tract infections (RTIs) per 7–10-year-old child, number of illness days, number of days with fever, number of days with cough, and number of days of antipyretic use and antibiotic use per child/per episode during the 4-month period of the trial.

	Verum (n = 18)	Placebo (n = 17)	Effect Size (Cohen’s *d*)	*p*-Value
Number of RTI episodes (per child)	2.61 ± 0.70	4.12 ± 1.83	0.9	0.01881
Number of illness days (per child/per episode)	2.94± 0.54	4.00 ± 0.50	2.0	0.00001
Number of days with fever (per child/per episode)	3.07 ± 1.60	3.36 ± 0.95	0.2	0.14580
Number of days with cough (per child/per episode)	4.47 ± 1.58	5.94 ± 1.14	1.1	0.00369
Number of days with rhinitis (per child/per episode)	5.34 ± 1.58	5.82± 1.74	0.3	0.13790
Number of days of antipyretic use (per child/per episode)	2.68 ± 1.25	3.42 ± 1.10	0.6	0.01273
Number of days of antibiotic use (per child/per episode)	3.22 ± 1.35	3.59 ± 1.80	0.2	0.09472

Data are expressed as means ± standard deviation. *p*-values for Mann–Whitney test for comparison of verum vs. placebo.

**Table 6:** Relative risk (RR) of 110 children to have one episode, ≥two episodes, or ≥three episodes of respiratory tract infections (RTIs) during the 4-month period of the trial.

	Verum (n = 56)	Placebo (n = 54)	RR 95% CI	*p*-Value
Number of children having one RTI episode	34/56	44/54	0.745 (0.583–0.953)	0.009
Number of children having ≥two < three RTI episodes	19/56	41/54	0.445 (0.306–0.677)	0.00005
Number of children having ≥three RTI episodes	7/56	32/54	0.211 (0.102–0.437)	0.00001

**Table 7:** Summary of adverse events (AEs).

	Verum (n = 56)	Placebo (n = 55)
**Total number of AEs reported (n)**	0	1
**AEs symptoms**		
– Headache	0	0
– Appearance of spots on the tongue	0	1
– Gastrointestinal disease	0	0
**Severity of AEs**		
– Mild	0	1
– Moderate	0	0
– Severe	0	0
**Serious Adverse Events (SAEs)**	none	none
**Withdrawals due to AEs**	0	1
**Relationship to study treatment**		
– Not related	0	1
– Possibly related	0	0
– Probably related	0	0
– Related	0	0

## Data Availability

The data presented in this study are available on request from the corresponding author. The data are not publicly available because dataset contains personal and sensitive information.

## Abbreviations

The following abbreviations are used in this manuscript:

RTIs	Respiratory tract infections
RR	Relative risk
CI	Confidence interval
WHO	World Health Organization
AOU	Azienda Ospedaliera Universitaria
CRO	Contract Research Organization
